# Coronary physiology before and after chronic total occlusion treatment: what does it tell us?

**DOI:** 10.1007/s12471-020-01470-6

**Published:** 2020-07-27

**Authors:** D. C. J. Keulards, P. J. Vlaar, I. Wijnbergen, N. H. J. Pijls, K. Teeuwen

**Affiliations:** 1 Catharina Hospital, Eindhoven, The Netherlands; 2 Eindhoven University of Technology, Eindhoven, The Netherlands

**Keywords:** Chronic total occlusion, Physiology, Fractional flow reserve, Coronary flow reserve, Percutaneous coronary intervention

## Abstract

Studies performed in the last two decades demonstrate that after successful percutaneous coronary intervention (PCI) of a chronically occluded coronary artery, the physiology of the chronic total occlusion (CTO) vessel and dependent microvasculature does not normalise immediately but improves significantly over time. Generally, there is an increase in fractional flow reserve (FFR) in the CTO artery, a decrease in collateral blood supply and an increase in FFR in the donor artery accompanied by an increase in blood flow and decrease in microvascular resistance in the myocardium supplied by the CTO vessel. Analogous to these physiological changes, positive remodelling of the distal CTO artery also occurs over time, and intravascular imaging can be helpful for analysing distal vessel parameters. Follow-up coronary angiography with physiological measurements after several weeks to months can be helpful and informative in a subset of patients in order to decide upon the necessity for treatment of residual coronary artery stenosis in the vessel distal to the CTO or in the contralateral donor artery, as well as in deciding whether stent optimisation is indicated. We suggest that such physiological guidance of CTO procedures avoids unnecessary overtreatment during the initial procedure, guides interventions at follow-up, and improves our understanding of what PCI in CTO means.

## Introduction

To answer the question as to what coronary physiology teaches us during and after percutaneous coronary intervention (PCI) of chronic total occlusion (CTO), it is important to emphasise that interventional treatment should be based upon knowledge and understanding of the underlying pathophysiology. Why does a patient suddenly present with angina caused by a CTO that has existed for years? How does a stenosis in the donor artery influence CTO blood supply? What kind of improvement can be expected following PCI?

For a long time, coronary physiology measurement was underappreciated in chronic coronary occlusions, but it has attracted more interest in recent decades [1, 2]. In most CTOs with viable distal myocardium, collateral blood supply is present and sufficient to maintain resting metabolism (hibernation) or even contraction at rest. This collateral circulation generally develops over several weeks to months and the extent of the collateral network depends on the acuity of the occlusion, the duration and frequency of ischaemia, genetic influence and co-morbid states, e.g. diabetes [3]. Lee et al. [3] also compared two groups of total occlusions, i.e. the acute occlusions during myocardial infarction and the chronic total occlusions. They noticed that the collateral flow assessed by coronary wedge pressure in relation to aortic pressure was higher in CTO than in acute occlusions.

Mostly, however, even in the presence of collaterals, frank ischaemia occurs in the CTO-supplied myocardium with increasing demand during stress/exercise [4, 5]. With viable myocardium (e.g. demonstrated by preserved wall motion at rest) and typical chest pain, additional ischaemia testing is not necessary and PCI of CTO can be planned. In cases of regional wall motion abnormalities in the territory of the CTO, other objective evidence of viability should be sought before making the decision to perform PCI [6]. This is more important because PCI of CTO is complex and target lesion failure is still much higher compared to non-CTO PCI [7]. Physiological measurements in the recanalised CTO artery, its dependent myocardium and donor artery may be helpful in optimising the procedure and might improve long-term clinical outcomes.

## The last two decades in physiology

With the evolution of intracoronary physiological evaluation, CTO vessels have also become a topic of interest and many studies have attempted to explain the complex physiology in CTO vessels and the dependent myocardium. CTO-vessel PCI has three major physiological effects: (1) effects on the CTO vessel and the dependent myocardium; (2) effects on the donor vessels; and (3) effects on the interaction between the two, i.e. the collateral circulation. Table 1 summarises a number of these studies categorised by the site of change and according to the technique used [3, 5, 8–18].

**Table 1:** Overview of human physiology studies, last two decades

Author	Technique	*n*	Baseline value	Value immediately after procedure	*p*-value	Value at follow-up	*p*-value	Interval to follow-up
**CTO vessel changes**								
*Coronary flow reserve (CFR) or Doppler velocity*								
Werner [8]	CFR Doppler + FFR	42		2.01		Same	n. s.	Immediate
Werner [9]	CFR Doppler + FFR	120		2.01		2.50	*<0.01*	5 months
Schumacher [10]	(CFR) PET	193	1.59			2.88	*<0.01*	3 months
Stuijfzand [11]	MBF (PET)	69	1.22			2.40	*<0.01*	3 months
Schumacher [12]	CFR (PET)	193	1.52			2.89	*<0.01*	3 months
*Fractional flow reserve (FFR)*								
Zimarino [13]	FFR	42	<0.5	>0.8	*<0.01*			Immediate
Sachdeva [5]	FFR	100	0.45	0.90	*<0.01*			Immediate
Lee [3]	FFR	74	0.48	0.86	*<0.01*	0.87	n. s.	12 months
Karamasis [14]	FFR	26		0.82		0.89	*<0.01*	4 months
*Absolute blood flow and resistance measurements (ml/min and Wood units)*								
Keulards [15]	Absolute flow/resistance	25		148		221	*<0.01*	2 months
**Donor vessel changes**								
*Coronary flow reserve (CFR) or Doppler velocity*								
Ladwiniec [16]	CFR Doppler + FFR	34	2.24	2.33	0.57			Immediate
*Instantaneous wave-free ratio (iFR)*								
Mohdnazri [17]	iFR	34	0.86	0.88	*<0.05*	0.90	*<0.05*	4 months
*Fractional flow reserve (FFR)*								
Sachdeva [18]	FFR	14	0.76	0.86	*<0.01*			Immediate
Ladwiniec [16]	FFR + Doppler	34	0.78	0.81	*<0.01*			Immediate
Mohdnazri [17]	FFR	34	0.76	0.75	0.267	0.79	*<0.05*	4 months
**Collateral circulation regression**								
*Collateral fractional flow reserve (FFR*_*coll*_*)*								
Zimarino [13]	FFR_coll_	42	0.42	0	*<0.01*			Immediate
Karamasis [14]	FFR_coll_	26	0.29	0.33	n. s.	0.18	*<0.01*	4 months

*CTO* chronic total occlusion, *MBF* myocardial blood flow, *PET* positron emission tomography, *n.* *s.* not significant

Roughly, these studies can be divided into three ‘groups’: the flow and velocity studies (coronary flow (velocity) reserve, CF(V)R); the pressure studies (fractional flow reserve (FFR), instantaneous wave-free ratio (iFR)); and absolute blood flow and resistance studies.

First, CFVR reflects the ratio between basal and hyperaemic coronary flow velocity, measured by a Doppler wire. This tells us something about the functionality of the whole coronary circulation, both epicardial and microcirculatory, but does not enable us to distinguish between these two compartments. It reflects blood flow increase during stress/exercise situations (or hyperaemia in the catheterisation laboratory). A value ≥2.0 is often considered to be normal, which means that basal coronary blood flow can double during hyperaemia. CFVR is used as an equivalent to coronary flow reserve (CFR), defined as the ratio of absolute hyperaemic blood flow to baseline blood flow. CFR can also be approximated by thermodilution using a bolus of saline to measure mean transit time at rest and during maximum hyperaemia. CFR measured by thermodilution is then represented by the ratio of mean transit times [19].

Studies with CFR or CFVR show the same trends: peak flow (velocity) increases directly after opening the CTO vessel [20] corresponding to a higher hyperaemic flow, and CF(V)R increases further over time, indicating further improvement of the myocardium and/or epicardial remodelling [9, 12].

Use of CF(V)R in clinical practice is limited because of the fact that coronary blood flow (velocity) is influenced by many factors, such as blood pressure, heart rate, vessel diameter, age and others. This is particularly due to difficulties in obtaining an unequivocal resting value. Consequently, there is a large variation in ‘normal’ values. Taking this into consideration, values can vary significantly in the same patient and over time. In addition, Doppler techniques are sensitive to patient motion, breathing, minimal position changes of the sensor and are as such operator-dependent.

**Fig. 1:**
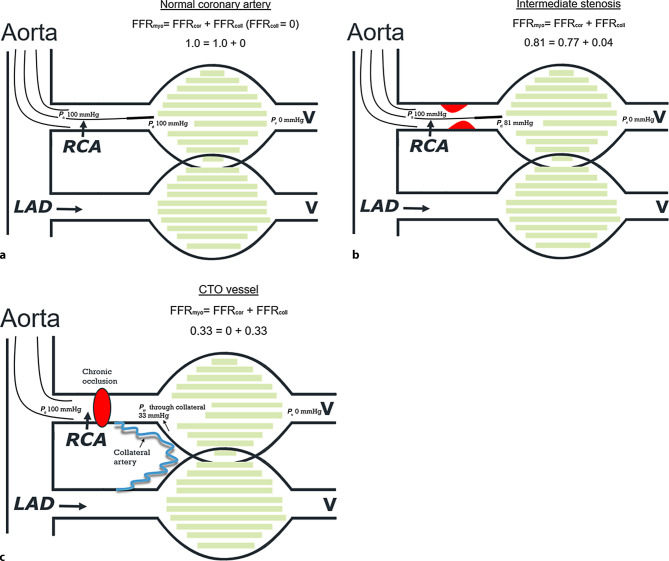
Fractional flow reserve (*FFR*) in a normal vessel (**a**), in an intermediate stenosis (**b**) and a chronic coronary total occlusion (*CTO*) (**c**). Generally, when using myocardial FFR (*FFR*_*myo*_), venous pressure (*P*_v_) is neglected and FFR is defined as the distal coronary to aortic pressure ratio (*P*_d_ */* *P*_a_) at maximum hyperaemia. When there is no stenosis (**a**) there is no pressure loss over the coronary artery, meaning *P*_a_ and *P*_d_ are equal and FFR is 1.0. In an intermediate stenosis (**b**) FFR is decreased due to the loss of pressure over the stenosis and the contribution of collateral blood flow is small. In the case of CTO (**c**) collateral flow becomes the predominant contributor to distal myocardial perfusion. The collateral contribution can be calculated separately by FFR_coll_ = *P*_w_ / *P*_a_, where *P*_w_ is wedge pressure. If there is elevated *P*_v_, this component should be measured separately. *FFR*_*cor*_ coronary FFR, *RCA* right coronary artery, *LAD* left anterior descending artery, *V* venous pressure

A second method used to investigate coronary physiology is based on pressure measurements. The FFR provides information specific to the epicardial stenosis. To measure FFR, a high-fidelity intracoronary pressure wire is used to record the distal coronary pressure (*P*_d_) during maximum hyperaemia. FFR is the ratio between *P*_d_ and aortic pressure (*P*_a_) and specifies the pressure loss across a stenosis (Fig. 1a). Normal FFR is close to 1.0 and a value >0.80 is considered to be non-ischaemic, which means that if less than 20% of coronary pressure is lost across the stenosis during hyperaemia, no ischaemia will occur (Fig. 1a).

**Fig. 2:**
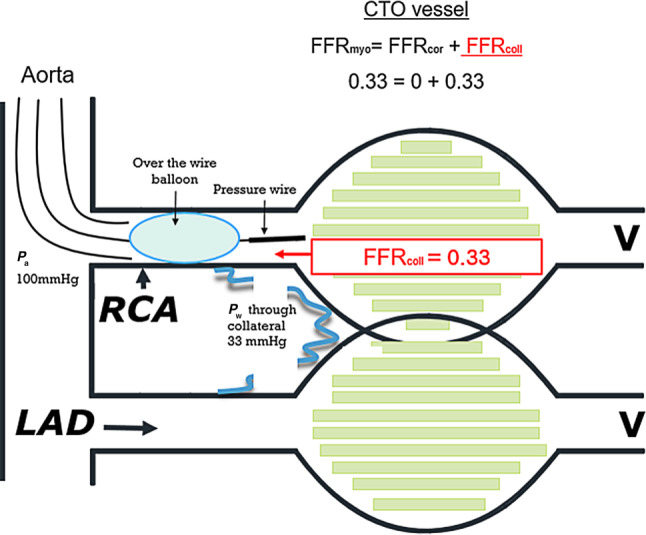
Collateral fractional flow reserve (*FFR*_*coll*_) assessment using coronary wedge pressure (*P*_*w*_). Example of how to assess *P*_w_. In order to evaluate the contribution of collateral vessels a pressure wire can be advanced into the just opened chronic total occlusion (*CTO*) vessel. Then a balloon is advanced over the pressure wire and inflated at low atmospheric pressure to occlude the vessel transiently (preferably within the just placed stent) and *P*_w_ is measured. FFR is still represented by the ratio of *P*_d_ (called now *P*_w_) and *P*_a_. When antegrade flow is 0, FFR consists exclusively of FFR_coll_. *FFR*_*myo*_ myocardial FFR, *FFR*_*cor*_ coronary FFR, *RCA* right coronary artery, *LAD* left anterior descending artery, *V* venous pressure

When using FFR in CTO physiology, it is important to realise that FFR as usually used (and represented by *P*_d_ / *P*_a_) represents the influence of a coronary stenosis on myocardial blood flow and that myocardial blood flow is the sum of coronary artery blood flow and collateral blood flow. Under normal circumstances, the role of collateral flow is negligible, but with severe stenosis and in particular in CTO, collateral flow becomes important. This can be quantified by FFR (originally called FFR_myocardium_) because it can be split up as: FFR_myocardium_ (FFR_myo_) = FFR_coronary_ (FFR_cor_) + FFR_collateral_ (FFR_coll_), and all of these three compartments can be assessed separately by pressure measurements (Fig. 1). To do so, measurement of coronary wedge pressure (during balloon occlusion of the coronary artery) is mandatory, as explained in Figs. 1 and 2.

**Fig. 3:**
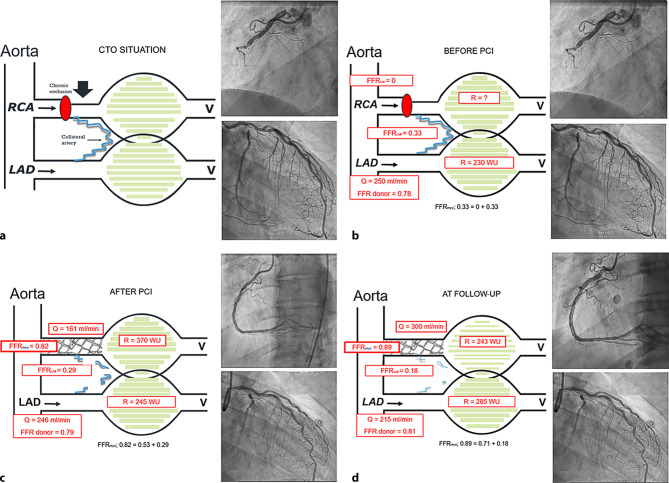
Example synthesising all angiographic and physiological measurements before and after percutaneous coronary intervention (*PCI*) and at follow-up. All values in the images are based on measurements in one selected patient. To date, no large studies have combined all measurements. **a** Schematic and angiographic situation before intervention with a chronic total occlusion (*CTO*) of the right coronary artery (*RCA*) and donor arteries from the left anterior descending (*LAD*) artery to the distal RCA. **b** Situation before PCI of the CTO when assessment of only collateral and donor vessel flow is possible. **c** Situation immediately after opening the CTO vessel. Now both assessment of antegrade flow and fractional flow reserve (*FFR*) of the CTO vessel and donor/collateral supply is possible. **d** Assessment of all values is possible. *FFR*_*cor*_ fractional flow reserve of the coronary artery, *FFR*_*coll*_ collateral fractional flow reserve, *FFR*_*myo*_ myocardial fractional flow reserve (= FFR_cor_ + FFR_coll_), *Q* absolute myocardial blood flow in ml/min, *R* microvascular resistance in Wood units (*WU*) (mm Hg · ml/min), *V* venous pressure

Lee et al. [3] and Karamasis et al. [14] showed that during follow-up measurements in the CTO vessel, FFR increased significantly over time. In the study by Karamasis et al., comprehensive FFR measurements were accompanied by FFR_coll_ assessment immediately after CTO PCI and at 16-week follow-up. The study showed that the increase of FFR_myo_ over time was accompanied by a significant reduction of collateral function as expressed by FFR_coll_ (Fig. 3c,d)_._ This was also in line with the earlier publication by Zimarino et al. [13], who showed that FFR_coll_ decreased significantly.

In addition to antegrade blood flow in the re-opened CTO artery, donor vessels have also been evaluated extensively ([14, 16–18, 21]; Fig. 3b–d). Most studies showed a slight increase in donor artery FFR and iFR directly after PCI. Longer-term follow-up studies showed a more pronounced increase in FFR of the donor artery, explained by the decrease of its perfusion territory. The late change in FFR also confirms delayed collateral regression.

Although FFR measurement before and after PCI is feasible and safe, it does not provide information on the function of the microvasculature. To make a better distinction between epicardial coronary artery function and microvascular function, CFR has been combined with FFR. ‘Low’ CFR (<2.0) and ‘high’ FFR (≥0.80) would indicate a normal epicardial artery but abnormal microvascular function. However, such combined measurements at best provide qualitative information but do not allow true quantification of microvascular function.

At this point, a relatively new and promising methodology to measure absolute coronary blood flow and microvascular resistance quantitatively comes into consideration [22–25]. The measurement of absolute blood flow and resistance uses a technique of thermodilution with continuous low-rate infusion of saline. The application of this established method is very easy, reproducible and without noticeable side-effects [22]. The measurements are performed during maximum hyperaemia induced by saline [26] using a multifunctional monorail infusion catheter as described and validated extensively (Rayflow, Hexacath, Paris, France) [23, 24, 27]. The method is based on the calculation of flow (*Q* in ml/min) and microvascular resistance (*R* in dyn · s/cm^−5^, mm Hg · l/min, or Wood units). The flow is calculated after continuous intracoronary infusion of saline at a chosen infusion rate creating maximum hyperaemia [26]. The distal coronary temperature (*T*) is measured using a normal pressure wire X (PWX, Abbott, Chicago, IL, USA). After reaching steady-state hyperaemia (mostly within 20 s) and measuring *T*, the pressure wire is pulled back into the Rayflow catheter to determine the infusion temperature of the saline (*T*_i_). Absolute blood flow is then calculated by dividing the infusion temperature by the distal temperature and multiplying it by the infusion rate of the pump, following the equation below:

$$Q_{\mathrm{b}}=1.08\,\frac{T_{\mathrm{i}}}{T}Q_{\mathrm{i}}$$

where *Q*_b_ is the flow in ml/min. The constant 1.08 relates to the difference between the specific heats and densities of blood and saline. *T*_i_ is the infusion temperature of the saline at the infusion holes of the Rayflow catheter and *T* is the distal coronary temperature measured by the pressure wire, both expressed as the difference to body temperature. *Q*_i_ is the infusion rate of the pump in ml/min.

Simplified this means that when *T* is 1°C below body temperature and *T*_i_ is 5°C below body temperature, the absolute blood flow is 5 times the set rate of saline infusion. So in this example, with a saline infusion rate of 20 ml/min, blood flow will be 108 ml/min. Microvascular resistance is calculated in analogy to Ohm’s law by dividing the distal pressure and flow.

All calculations for absolute flow and resistance, explained above, are automatically made using the Coroventis software program (Coroventis, Uppsala, Sweden). The accuracy and reproducibility of these absolute flow and resistance measurements have been validated extensively, and the procedure can be performed safely and quickly following regular FFR measurement [22, 25].

Since the patient acts as her/his own control during follow-up procedures, accurate evaluation of blood flow and resistance at follow-up of both the epicardial artery and the microvasculature is possible. In an exploratory study by Keulards et al. [15] these measurements were performed in both the CTO and donor vessel territory and showed a significant increase in hyperaemic coronary blood flow and improvement in microvascular function a few weeks following the procedure. Maximum blood flow in the CTO artery increased by 49% and microvascular resistance decreased by 29% at follow-up compared to the situation immediately after the index procedure (Fig. 3d). At the same time, distal CTO vessel diameter increased. It appears that the increase in coronary flow is a combination of two factors, i.e. decrease in microvascular resistance, reflecting further improvement of the microcirculation, and decrease in distal epicardial resistance with positive remodelling of the distal CTO artery. Fig. 3 summarises all the physiological changes mentioned above.

## What does integrated coronary physiology tell us?

The studies mentioned above demonstrate that after successful PCI of a chronically occluded coronary artery, blood flow in the CTO vessel and microvascular function do not normalise immediately but improve significantly over time. This is reflected by an increase in absolute blood flow and FFR of the CTO artery, decrease in microvascular resistance, and regression of collaterals with decrease in FFR_coll_. As a corollary, corresponding changes in the donor artery can often be observed. The FFR of the major donor vessel may increase, reflecting decrease of collaterals.

Being aware of such anticipated physiological and anatomical changes may have three important consequences for interventional decision-making. First, the diameter of the vessel distal to the occlusion directly after opening the CTO is still negatively remodelled, which may mislead the operator into taking a smaller stent for CTO treatment even after an optimal dosage of nitroglycerin. Second, due to negative remodelling of the distal segment, a distal stenosis can seem significant. This can lead to longer stents and even more restenosis [28]. And thirdly, FFR of an intermediate stenosis in the donor artery with values slightly below the ischaemic thresholds before the CTO artery is opened may increase over time, thereby avoiding PCI of that intermediate lesion. In the current ESC guidelines [6] it is suggested that the donor vessel is stented first. But when CTO PCI is planned anyway, this strategy may be changed if there are ischaemic lesions in the donor artery. Larger trials are required to add further support to this strategy, but such adjustments may decrease the number of stents, the rate of restenosis over time and improve our knowledge and patient care.

Previous research reflected on a sort of ‘vascular wall hibernation’ of the distal coronary segments of a recanalised CTO failing to show a response to endothelium-dependent and endothelium-independent stimuli [20, 29]. The increase in hyperaemic absolute coronary blood flow over time can be partially explained by the above findings and strongly supports improved distal coronary artery wall function.

## Role of intracoronary imaging

For follow-up of the aforementioned positive remodelling of the distal CTO artery over time, both intravascular ultrasound (IVUS) and optical coherence tomography (OCT) can be helpful for measuring ‘true distal vessel’ parameters. In daily practice, IVUS is preferred to OCT for PCI guidance in CTO cases, because using OCT increases the risk of dissection before stenting and requires high volumes of contrast. It is important to realise, however, that even when intracoronary imaging is used, vessel diameters can still be underestimated due to chronic negative remodelling. Also, using IVUS it has been shown that positive remodelling occurs over time [30]. Intracoronary imaging at follow-up can also be used to decide whether the stents have been undersized and need to be optimised [28, 31]. We have learned that as a rule of thumb stents with a diameter that is 0.4 mm larger should be chosen in cases of CTO to diminish late stent malapposition, but when there is doubt regarding sizing a follow-up invasive examination with re-evaluation of stent size can be useful.

## The future

The number of large cohort studies in CTO is still limited. Presently, the IMPACT-CTO 2 trial is being performed (ClinicalTrials.gov identifier: NCT03830853), in which all relevant physiological indices (FFR, FFR_coll_, absolute flow and resistance) are assessed both in the donor vessel and in the CTO artery immediately after PCI and after 12 weeks. This is combined with intracoronary imaging, to account for remodelling of the distal CTO artery. These measurements will likely provide an integrated and more complete insight into coronary physiology and anatomy after PCI of CTO arteries and can be helpful in planning a CTO follow-up procedure.

## References

[1] Konstantinidis NV, Werner GS, Deftereos S, Di Mario C, Galassi AR, Buettner JH (2018). Temporal trends in chronic total occlusion interventions in Europe. Circ Cardiovasc Interv.

[2] Tajti P, Karmpaliotis D, Alaswad K, Jaffer FA, Yeh RW, Patel M (2018). The hybrid approach to chronic total occlusion percutaneous coronary intervention: update from the PROGRESS CTO Registry. JACC Cardiovasc Interv.

[3] Lee JH, Kim C-Y, Kim N, Jang SY, Bae MH, Yang DH (2017). Coronary collaterals function and clinical outcome between patients with acute and chronic total occlusion. JACC Cardiovasc Interv.

[4] Stuijfzand WJ, Driessen RS, Raijmakers PG, Rijnierse MT, Maeremans J, Hollander MR (2017). Prevalence of ischaemia in patients with a chronic total occlusion and preserved left ventricular ejection fraction. Eur Heart J Cardiovasc Imaging.

[5] Sachdeva R, Agrawal M, Flynn SE, Werner GS, Uretsky BF (2014). The myocardium supplied by a chronic total occlusion is a persistently ischemic zone. Catheter Cardiovasc Interv.

[6] 2018 ESC/EACTS Guidelines on myocardial revascularization. Eur Heart J. [cited 2020 Mar 16]. Available from: https://academic.oup.com/eurheartj/article/40/2/87/5079120. Accessed May 2020.

[7] Azzalini L, Carlino M, Bellini B, Marini C, Pazzanese V, Toscano E (2020). Long-term outcomes of chronic total occlusion recanalization versus percutaneous coronary intervention for complex non-occlusive coronary artery disease. Am J Cardiol.

[8] Werner GS, Ferrari M, Richartz BM, Gastmann O, Figulla HR (2001). Microvascular dysfunction in chronic total coronary occlusions. Circulation.

[9] Werner GS, Emig U, Bahrmann P, Ferrari M, Figulla HR (2004). Recovery of impaired microvascular function in collateral dependent myocardium after recanalisation of a chronic total coronary occlusion. Heart.

[10] Schumacher SP, Stuijfzand WJ, Driessen RS, van Diemen PA, Bom MJ, Everaars H (2019). Impact of specific crossing techniques in chronic total occlusion percutaneous coronary intervention on recovery of absolute myocardial perfusion. Circ Cardiovasc Interv.

[11] Stuijfzand WJ, Biesbroek PS, Raijmakers PG, Driessen RS, Schumacher SP, van Diemen P (2017). Effects of successful percutaneous coronary intervention of chronic total occlusions on myocardial perfusion and left ventricular function. EuroIntervention.

[12] Schumacher SP, Kockx M, Stuijfzand WJ, Driessen RS, van Diemen PA, Bom MJ (2019). Relationship between extent of ischaemic burden and changes in absolute myocardial perfusion after chronic total occlusion percutaneous coronary intervention. EuroIntervention.

[13] Zimarino M, Ausiello A, Contegiacomo G, Riccardi I, Renda G, Di Iorio C (2006). Rapid decline of collateral circulation increases susceptibility to myocardial ischemia: the trade-off of successful percutaneous recanalization of chronic total occlusions. J Am Coll Cardiol.

[14] Karamasis GV, Kalogeropoulos AS, Mohdnazri SR, Al-Janabi F, Jones R, Jagathesan R (2018). Serial fractional flow reserve measurements post coronary chronic total occlusion percutaneous coronary intervention. Circ Cardiovasc Interv.

[15] Keulards DCJ, Karamasis GV, Alsanjari O, Demandt JPA, Van’t Veer M, Zelis JM (2020). Recovery of absolute coronary blood flow and microvascular resistance after chronic total occlusion percutaneous coronary intervention: an exploratory study. J Am Heart Assoc..

[16] Ladwiniec Andrew, Cunnington Michael S., Rossington Jennifer, Mather Adam N., Alahmar Albert, Oliver Richard M., et al. Collateral donor artery physiology and the influence of a chronic total occlusion on fractional flow reserve. Circ Cardiovasc Interv. 2015;8:e002219. 10.1161/CIRCINTERVENTIONS.114.002219 25805570

[17] Mohdnazri SR, Karamasis GV, Al-Janabi F, Cook CM, Hampton-Till J, Zhang J (2018). The impact of coronary chronic total occlusion percutaneous coronary intervention upon donor vessel fractional flow reserve and instantaneous wave-free ratio: implications for physiology-guided PCI in patients with CTO. Catheter Cardiovasc Interv.

[18] Sachdeva R, Agrawal M, Flynn SE, Werner GS, Uretsky BF (2013). Reversal of ischemia of donor artery myocardium after recanalization of a chronic total occlusion. Catheter Cardiovasc Interv.

[19] Fearon WF, Balsam LB, Farouque HMO, Caffarelli AD, Robbins RC, Fitzgerald PJ (2003). Novel index for invasively assessing the coronary microcirculation. Circulation.

[20] Brugaletta S, Martin-Yuste V, Padró T, Alvarez-Contreras L, Gomez-Lara J, Garcia-Garcia HM (2012). endothelial and smooth muscle cells dysfunction distal to recanalized chronic total coronary occlusions and the relationship with the collateral connection grade. JACC Cardiovasc Interv.

[21] Tigen K, Durmuş E, Sari I (2014). Recanalization of a total occlusion with marked retrograde collateral supply: impact of collateral circulation on fractional flow reserve measurements of donor artery. J Invasive Cardiol.

[22] Keulards DCJ, Van ’t Veer M, Zelis JM, El Farissi M, Zimmermann FM, de Vos A (2020). Safety of absolute coronary flow and microvascular resistance measurements by thermodilution. EuroIntervention.

[23] Aarnoudse W, Van’t Veer M, Pijls NHJ, Ter Woorst J, Vercauteren S, Tonino P (2007). Direct volumetric blood flow measurement in coronary arteries by thermodilution. J Am Coll Cardiol.

[24] Veer M van’t, Adjedj J, Wijnbergen IF, Toth GG, Rutten MCM, Barbato E, et al. Novel monorail infusion catheter for volumetric coronary blood flow measurement in humans. Eurointervention. 12(6):701–7. 10.4244/EIJV12I6A114. 10.4244/EIJV12I6A114 27542781

[25] Xaplanteris P, Fournier S, Keulards DCJ, Adjedj J, Ciccarelli G, Milkas A (2018). Catheter-based measurements of absolute coronary blood flow and microvascular resistance: feasibility, safety, and reproducibility in humans. Circ Cardiovasc Interv.

[26] De Bruyne B, Adjedj J, Xaplanteris P, Ferrara A, Mo Y, Penicka M, et al. Saline-induced coronary hyperemia: mechanisms and effects on left ventricular function. Circ Cardiovasc Interv. 2017;10: 10.1161/CIRCINTERVENTIONS.116.004719. 10.1161/CIRCINTERVENTIONS.116.004719 28400462

[27] Everaars H, de Waard GA, Schumacher SP, Zimmermann FM, Bom MJ, van de Ven PM (2019). Continuous thermodilution to assess absolute flow and microvascular resistance: validation in humans using [^15^O]H_2_O positron emission tomography. Eur Heart J.

[28] Zivelonghi C, Suttorp MJ, Teeuwen K, van Kuijk JP, van der Heyden JAS, Eefting FD (2018). Clinical implications of distal vessel stenosis after successful coronary chronic total occlusion recanalization. JACC Cardiovasc Interv.

[29] Galassi AR, Tomasello SD, Crea F, Costanzo L, Campisano MB, Marzá F (2012). Transient impairment of vasomotion function after successful chronic total occlusion recanalization. J Am Coll Cardiol.

[30] Saito S, Maehara A, Yakushiji T, Dohi T, Kobayashi N, Song L (2016). Serial intravascular ultrasound findings after treatment of chronic total occlusions using drug-eluting stents. Am J Cardiol.

[31] Teeuwen K, Spoormans EM, Bennett J, Dubois C, Desmet W, Ughi GJ (2017). Optical coherence tomography findings: insights from the “randomised multicentre trial investigating angiographic outcomes of hybrid sirolimus-eluting stents with biodegradable polymer compared with everolimus-eluting stents with durable polymer in chronic total occlusions” (PRISON IV) trial. EuroIntervention.

